# Effects of daidzein on rat ovary against ischemia-reperfusion

**DOI:** 10.1590/acb384423

**Published:** 2023-10-30

**Authors:** Veysel Toprak, Senem Alkan Akalın, Ece Öcal, Yunus Çavuş, Engin Deveci

**Affiliations:** 1 Eyyübiye Education and Research Hospital – Department of Gynecology and Obstetrics – Şanlıurfa – Turkey.; 2 Division of Gynecology and Obstetrics – Private Medical Practice – Bursa – Turkey.; 3 Division of Perinatology – Private Medical Practice – Diyarbakir – Turkey.; 4 Diyarbakır Bower Hospital – Department of Gynecology and Obstetrics – Diyarbakır – Turkey.; 5 Dicle University – Faculty of Medicine – Department of Histology and Embryology – Diyarbakır – Turkey.

**Keywords:** Ovary, Ischemia, Reperfusion, Pathology, Histology

## Abstract

**Purpose:**

Our aim was to investigate protective effects of daidzein treatment on ischemia-reperfusion (I/R) injury-induced ovarian tissue by immunohistochemical techniques.

**Methods:**

Thirty Sprague Dawley female rats were categorized into three groups as sham, I/R group, and I/R+daidzein groups. Bloods were analyzed for malondialdehyde (MDA), glutathione peroxidase (GSH), and myeloperoxidase (MPO), and ovaries were processed for histological tissue protocol.

**Results:**

Both MDA and MPO values were increased in I/R group compared to sham and I/R+daidzein groups. GSH content was increased in I/R+daidzein group compared to I/R groups. In I/R group, theca and follicular cells were degenerated with apoptosis and dilatation and congestion, edema. In I/R+daidzein group, daidzein improved pathologies. In the I/R group, Bax expression was positive with follicular cells, granulosa cells and inflammatory cells. In the I/R+daidzein group, positive Bax reaction was observed in the epithelial, antral, and inflammatory cells. In I/R group, Bcl-2 reaction was in germinative epithelial cells, cells of antral follicle. In the I/R+daidzein group, Bcl-2 expression level was reduced after daidzein treatment.

**Conclusions:**

After the I/R procedure, ovarian cells and follicles were degenerated with apoptosis and inflammation. After daidzein treatment, Bax and Bcl-2 signal were decreased. It was observed that daidzein stopped the apoptotic process.

## Introduction

Ischemia is one of the most common causes of cell damage in clinical complications defined as decrease in the amount of blood flowing into the tissue. Once the cell damage is irreversible, re-initiation of blood flow can heal cells. However, in some cases, re-flow of blood may accelerate injury process, leading to tissue damage during restoration of blood period^1^^,^ 2. Ischemia-reperfusion (I/R) injury is related to many clinical disorders such as myocardial infarction, stroke, vascular diseases, organ transplantation, and shock. During I/R damage, reactive oxygen radicals are released, complement system is activated, leukocyte-endothelial cell adhesion is increased^3^^,^ 4.

Ovarian torsion is the rotation of the peduncle of the ovarian arterial and venous vessels in its own axis in a partial or full turn, preventing blood flow. Ovarian torsion is seen in 2.7% of gynecological emergencies. In terms of age groups, although it covers all age groups from female fetuses to menopause, it is observed with a higher rate in women of reproductive age^5^^,^ 6. The etiology of ovarian torsion is not fully known, but most cases have functional cysts or neoplasms of the ovary. Delay or error in diagnosis may cause the patient to lose his ovary or decrease follicle reserve^7^. When clinical signs of acute abdomen (peritoneal irritation) are present, most cases undergo diagnostic laparotomy or laparoscopy. However, the clinical findings are vague in most cases of torsion. Consequently, delays occur in diagnosis and treatment^8^.

Daidzein is a phytoestrogen isoflavone found in soybeans and other legumes. The chemical composition of daidzein is similar to mammalian estrogens and may be useful for a dual purpose by substituting/inhibiting with the estrogen and estrogen receptor (ER) complex. Therefore, daidzein exerts protective effects against numerous diseases, particularly those associated with estrogen control, such as breast cancer, diabetes, osteoporosis, and cardiovascular disease^9^. However, daidzein also has other ER-independent biological activities, such as oxidative damage reduction, acting as an antioxidant, immunomodulatory as an anti-inflammatory agent, and regulation of apoptosis directly linked to its potential anticancer effects^10^.

Apoptosis is a programmed cell death that occurs in multicellular organisms. apoptosis takes place in tissue homeostasis, embryonic growth, and immunity. Bcl-2 protein family either promote or inhibit apoptosis by regulating pro-apoptotic proteins and anti-apoptotic proteins^11^. The balance between anti-apoptotic and pro-apoptotic protein expressions determines the outcome of. Bax is a protein that stimulates apoptosis and when expressed causes cytochrome c to be released from the mitochondria^12^.

In this study, we aimed to investigate impacts of daidzein on ovarian tissue against I/R injury by analyzing Bax and Bcl-2 immunoexpression.

## Methods

### Animals and experimental procedure

All experiments performed in this study were approved by the Ethics Committee for Animal Experimentation. Thirty Sprague Dawley female rats (weighing 200–250 g) were bought and housed in separate cages at 23 ± 2°C, 12 hours light/12 hours dark period at 45–55% humidity and were fed with standard pellet and water. Estrous cycles of rats were determined by vaginal smear taken at 6–12-hour intervals. After cell examination under microscope, 30 female rats in estrous cycle were included in the experiment. Before starting the experimental procedure, 90-mg/kg intramuscular ketamine hydrochloride (Ketalar; Pfizer, Istanbul, Turkey) and 8-mg/kg xylazine (Rompun; Bayer, Istanbul, Turkey) were given for general anesthesia. Rats were divided into three groups (10 rats per group), and the following procedures was applied to the groups:

- Sham group: No treatment was applied to animals. Only the abdomen was opened with a surgical protocol, and the abdominal folds closed without any further intervention;
- I/R group: The abdominal area was opened with a 2-cm midline incision. Ischemia was created for 3 hours with a disposable Bulldog clamp on ovarian tissues. Then, the ovaries were placed back to their normal positions, in their anatomical location, and the blood flow was reperfused for 3 hours;
- I/R+daidzein group: Ovaries were allowed to ischemia-reperfusion injury. The abdomen was closed without any further intervention. One hour after starting the protocol, 20-mg/kg daidzein was introduced to rats via oral route with oral gavage.

### Biochemical analysis

Malondialdehyde (MDA) levels and glutathione peroxidase (GSH) activities were determined in the ovaries of each rat, and the average values of each group were calculated. Each ovary sample was prepared as a 10% homogenate (according to weight) in 0.9% saline using a homogenizer on ice. Then, the homogenate was centrifuged at 2,000 rpm for 10 min, and the supernatant was collected. MDA levels were determined using the double heating method of Draper and Hadley^13^. MDA values were expressed as nanomoles per gram (nmol/g) of wet tissue. GSH activity was measured by the method of Paglia and Valentine^14^. An enzymatic reaction was initiated by the addition of hydrogen peroxide (H_2_O_2_) to a tube that contained reduced nicotinamide adenine dinucleotide phosphate, reduced glutathione, sodium azide, and glutathione reductase. The change in absorbance at 340 nm was monitored by spectrophotometry. Data were expressed as U/g protein. Myeloperoxidase (MPO) activity in tissues was measured by a procedure similar to that described by Hillegas et al.^15^. MPO is expressed as U/g tissue.

*Histological tissue processing*

Ovarian tissues were fixed in formaldehyde solution for 24 hours. Ovarian tissues were passed through ascending alcohol series and incubated in paraffin wax. Tissues samples were embedded in paraffin blocks. Five-micron sections were cut from the paraffin blocks with a rotary microtome and stained with hematoxylin-eosin (H&E) dye to examine the tissue histopathology. Follicular degeneration, inflammation, and hemorrhage parameters were evaluated. Ovarian tissues were processed for further immunohistochemical examination^16^.

### Immunohistochemistry protocol

Sections were deparaffinized in xylene and brought to distilled water. Endogenous peroxidase activity was blocked in 0.1% hydrogen peroxide (catalogue #TA-015-HP, Thermo Fisher Scientific, United States of America) for 20 min. Ultra V block (TA-125-UB, Thermo Fisher Scientific, United States of America) was applied for 10 min. Tissue sections were incubated with anti-Bcl2 antibody (cat:ab59348, Abcam, United States of America) and anti-Bax antibody (cat: ab32503, Abcam, United States of America) overnight. The sections were washed three times for 5 min in phosphate buffer solution (PBS) and then incubated with biotinylated secondary antibody for 25 min. After washing with PBS, streptavidin peroxidase (TA-125-SP, Thermo Fisher Scientific, United States of America) was applied to the sections for 25 min. The sections were washed three times for 5 min in PBS. Diaminobenzidine (TA-125-DB, Thermo Fisher Scientific, United States of America) was applied to the sections for up to 15 min as a chromogen. The control slides were prepared using the same procedure, without primary antibodies. Counterstaining was done using Harris’s hematoxylin for 45 second, dehydrated through ascending alcohol and cleared in xylene. The slides were mounted with Entellan (lot: 107961, Sigma-Aldrich, St. Louis, MO, United States of America) and examined under a light microscope (Olympus, Germany)^17^.

### Statistical analysis

For statistical analysis, IBM Statistical Package for the Social Sciences (SPSS) Statistics 25.0 (IBM Inc, Chicago, IL, United States of America) was used with a computer program. First, normality tests were applied to the data, and it was checked whether the data were normally distributed. Kruskal–Wallis’ test (non-parametric) was used for comparison between independent groups, and if there were a difference, Mann Whitney’s U test was used for paired comparisons. The data of this study are given as mean ± standard error. A value of P < 0.05 in all tests was considered statistically significant.

## Results

**Figure 1:**
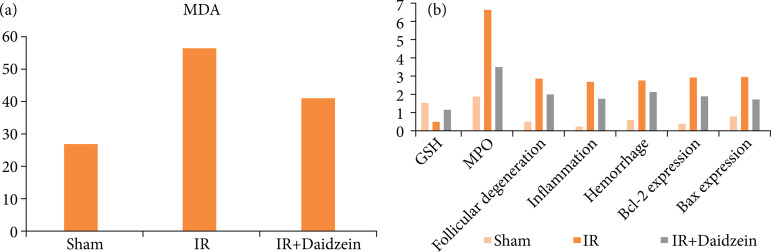
Biochemical parameters (MDA, GSH and MPO) and histological scores of ovarian tissues in sham, IR and IR+Daidzein groups.. **(a)** MDA values of sham, I/R and I/R+daidzein groups. **(b)** GSH, MPO and histological scoring for follicular degeneration, inflammation, hemorrhage, Bcl-2 and Bax expression in sham, I/R and I/R+daidzein groups.

Statistical analysis of biochemical and histochemical parameters was shown in Table 1 and Fig. 1. MDA and MPO levels and Bcl-2 and Bax expression were higher in I/R group than in sham group. Histological scores of follicular degeneration, inflammation, and hemorrhage were significantly higher in I/R group than in sham group. GSH content was significantly lower in the I/R group compared to sham group. After daidzein treatment, MDA and MPO levels were decreased in I/R+daidzein group compared to I/R group, and this decrease was statistically significant. Similarly, GSH content was statistically increased in I/R+daidzein group compared to I/R group. Histological scores of follicular degeneration, inflammation, and hemorrhage were decreased in I/R+daidzein group compared to I/R group. Bcl-2 and Bax expression were significantly reduced in I/R+daidzein group compared to I/R group.

**Table 1:** Biochemical and histological parameters of sham, I/R and I/R+daidzein groups.

Parameter	Groups	n	Median (min–max)	Mean rank	P-value
Malondialdehyde	Sham	10	25.12 (12.34–47.58)	10.50	0.001* 0.001**
	IR	10	54.69 (31.45–65.08)	25.50	
	I/R+daidzein	10	40.05 (20.98–50.39)	15.50	
Glutathione peroxidase	Sham	10	1.57 (1.22–1.93)	18.75	0.001* 0.001**
	I/R	10	0.42 (0.23–0.73)	10.25	
	I/R+daidzein	10	1.11 (0.88–1.63)	15.80	
Myeloperoxidase	Sham	10	1.89 (1.34–4.23)	7.50	0.001* 0.001**
	I/R	10	6.58 (3.93–8.83)	25.50	
	I/R+daidzein	10	3.47 (2.03–7.34)	16.50	
Follicular degeneration	Sham	10	0.50 (0.00–1.00)	15.00	0.001* 0.001*
	I/R	10	3.00 (2.00–3.00)	25.50	
	I/R+daidzein	10	2.00 (1.00–3.00)	20.70	
Inflammation	Sham	10	0.00 (0.00–1.00)	14.75	0.001** 0.001*
	I/R	10	3.00 (2.00–3.00)	23.50	
	I/R+daidzein	10	2.00 (1.00–3.00)	20.80	
Hemorrhage	Sham	10	0.00 (0.00–1.00)	10.80	0.001* 0.001*
	I/R	10	3.00 (2.00–3.00)	18.70	
	I/R+daidzein	10	2.00 (1.00–3.00)	15.50	
Bax expression	Sham	10	0.50 (0.00–1.00)	12.90	0.001** 0.001*
	I/R	10	3.00 (2.00–2.00)	25.75	
	I/R+daidzein	10	1.50 (1.00–3.00)	18.00	
Bcl-2 expression	Sham	10	1.00 (0.00–1.00)	13.50	0.001* 0.001**
	I/R	10	3.00 (2.00–3.00)	20.45	
	I/R+daidzein	10	1.50 (1.00–3.00)	15.55	

I/R: ischemia-reperfusion;

* sham vs. I/R;

** I/R vs. I/R+daidzein.

Source: Elaborated by the authors.

I/R: ischemia-reperfusion; MDA: malondialdehyde GSH: glutathione peroxidase; MPO: myeloperoxidase. Source: Elaborated by the authors.

### H&E findings

**Figure 2:**
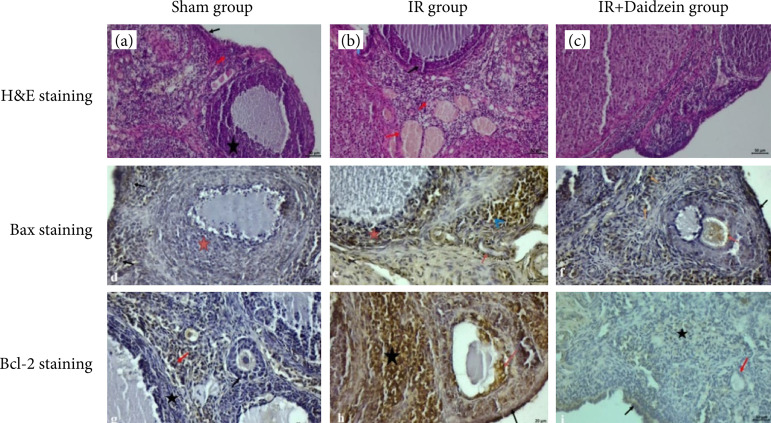
Histological staining of ovarian sections. Hematoxylin and eosin staining. **(a)** Sham group: smooth-looking germinative epithelium (black arrow) and primordial follicle (red arrow), smooth corpus luteum and stromal area (star). **(b)** IR group: degenerative changes in theca and granulosa cells in the corpus lutea of the antral follicle (black arrow), loss of structural integrity in the germinal epithelium (blue arrow), dilatation, congestion, and edema in the connective tissue areas (red arrow). **(c)** IR+daidzein group: the epithelial structure is smooth (black arrow), regular arrangement of granulosa cells in the corpus luteum (orange arrow), decrease in the area of inflammation (star). **(d)** Bax immunostaining group / sham group: positive Bax expression in some cells under the germinative epithelium (black arrows), negative Bax expression in granulosa cells (asterisk). **(e)** IR group: positive Bax expression in granulosa cells (asterisk), vessel endothelium (red arrow) and inflammation cells (blue arrowhead). **(f)** IR+Daidzein group: positive Bax reaction in germinative epithelium (black arrow), some cells in the stromal area (orange arrow), antral follicle cumulus oophorus (red arrow). **(g)** Bcl-2 immunostaining group / sham group: negative Bcl-2 expression in cells in preantral and antral follicle (black arrow), granulosa and theca cells in corpus luteum (star), and Bcl-2 reaction in base macrophage cells in stromal region (red arrow) moderate positivity. **(h)** IR group: Bcl-2 reaction positive in germinative epithelium (black arrow), antral follicle cumulus cells (red arrow), stromal area (star). **(i)** IR+daidzein group: positive Bcl-2 expression in germinal epithelial cells (black arrow), inflammatory cells (star) and negative Bcl-2 expression in granulosa cells of follicles (red arrow).

In the sham group, it was observed that the germinative epithelium was composed of prismatic epithelium with a slight protrusion towards the lumen, and the primordial and primary follicles exhibited a regular structure towards the lower sides. It was observed that the corpus luteum structure was smooth, and granulosa cells rich in chromatin were prominent. It was determined that the connective tissue cells diffusely spread in the stromal area had a regular structure (Fig. 2a). In I/R group, degenerative changes were observed in the theca and granulosa cells in the inner part of the corpus luteal structure of the antral follicle whose oocyte was expelled. In addition, the structural integrity was lost with apoptotic changes in the germinal epithelium. Dilatation and congestion were evident in the connective tissue areas outside the follicles, and edema was observed in places outside the vessel. It was determined that degenerative structures began to increase, especially as the cellular structures began to get smaller and smaller.

IR: ischemia and reperfusion. Source: Elaborated by the authors.

Apart from these changes, it was seen that there was an increase in mononuclear cell infiltration (Fig. 2b). In I/R+daidzein group, it was observed that the epithelial structure was smooth, protruding towards the lumen, and cilia were prominent. Although a regular arrangement was observed in the primordial cells under the epithelium, it was observed that some preantral and antral follicles regained their structural integrity. Although improvements were observed in the connective tissue structure in between, a decrease was verified in the inflammation structure. It was seen that the granulosa cells in the corpus luteum structure also showed a regular arrangement (Fig. 2c).

### Bax findings

In the sham group, positive Bax reaction was observed in some cells under the germinative epithelium, but completely negative Bax expression was observed in the granulosa cells in the area where the corpus luteum structure is located. In general terms, negative Bax expression was observed in the cells in both preantral and antral follicles in the stromal region (Fig. 2d). In the I/R group, Bax expression was also positive in the expanding vessel endothelium, especially in the granulosa cells of the corpus luteum. Bax expression was also found to be positive in the inflammation cells in between (Fig. 2e). In I/R+daidzein group, some cells showed a positive Bax reaction, especially in the epithelial regions, but a positive Bax reaction was observed in some cellular structures left from the zona pellucida in the antral follicle, where the oocyte was excreted, especially in cumulus cells. In the stromal region, cells with a positive Bax reaction were observed in the form of small inflammatory groups in between (Fig. 2f).

### Bcl-2 findings

In the sham group, it was observed that Bcl-2 expression was negative in the cells located in the preantral and antral follicles, as well as in the granulosa and theca cells around the corpus luteum. However, moderate Bcl-2 expression was observed in macrophage cells in some areas of the stromal region (Fig. 2g). In the I/R group, Bcl-2 immunohistochemical examination revealed positive Bcl-2 reaction in germinative epithelial cells. Again, it was observed that the Bcl-2 reaction was positive in the cumulus cells together with the degenerative cells in the antral follicle, which had discarded the developed oocyte 2. Similarly, Bcl-2 reaction was found positive in some preantral follicle structures in between. Bcl reaction was also positive in a small number of groups in the stromal area (Fig. 2h). In the I/R+daidzein group, Bcl-2 expression was positive in some germinal epithelial cells and inflammatory cells. Generally, negative Bcl-2 expression was observed in the connective tissue cells in the stroma and in the granulosa cells of the follicles (Fig. 2i).

## Discussion

In this experimental study, we studied effects of daidzein treatment in rat ovaries against I/R injury. We studied biochemical parameters MDA, GSH and MPO levels. MDA is a determinant of lipid peroxidation and provides the emergence of free radical. It can trigger various defense mechanisms with tissue damage caused by the production of reactive oxygen species.

One of the primary defense mechanisms is GSH. GSH is among the important components of intracellular protective mechanisms against a variety of harmful stimuli, including oxidative stress. MPO activity is an enzyme that explains the presence of neutrophils^18^^,^ 19. In a study on hepatic I/R injury in rats, they found that the I/R procedure with effect of grape seed extract caused a significant decrease in hepatic GSH, significant increases in MDA level and MPO activity. According to their findings, grape seed extract reduced I/R-induced organ damage thanks to its ability to balance oxidant-antioxidant status, inhibit neutrophil infiltration and regulate the release of inflammatory mediators^18^. Yapca et al.^20^ studied ovarian I/R and found that the levels of the oxidant parameters MDA and MPO were significantly higher in I/R group than control group, while the levels of the antioxidant parameters GSH were significantly lower in I/R group than in control group. Nayki et al.^21^ performed I/R in rat ovaries and recorded that MDA levels were increased in I/R group compared to control group. Another study on ovarian I/R showed that MDA level were the highest in the I/R group, and superoxide dismutase (SOD) and GSH activities were the lowest in I/R injured group^22^. In this study, MDA and MPO level were increased, and GSH content was decreased after I/R injury in I/R group compared to control group. Administration of daidzein lowered MDA and MPO level and elevated GSH content in I/R+daidzein group compared to IR group (Table 1, Fig. 1).

Apoptosis is a mode of programmed cell death that involves many molecular steps. Many pro-apoptotic and anti-apoptotic molecules take role in the apoptotic process. Bcl-2 protein family directly regulates intrinsic apoptosis and interacts mitochondrial membranes. The Bcl-2 protein itself is anti-apoptotic, although the other subgroup Bax protein is pro-apoptotic protein^23^^,^ 24. Kale et al.^23^ examined how post-translational modifications and different intracellular localizations alter the mechanisms of apoptosis regulation by BCL-2 family proteins. They concluded that successful therapeutic intervention of mitochondrial membrane permeability regulation in human disease requires an understanding of the factors that mediate major binding interactions between Bcl-2 family proteins in cells^23^.

On the other hand, a review study focused on the importance of BcL-2 for modulation of apoptosis at the mitochondrial level, its potential as a therapeutic target for hematological malignancies, and the results obtained with selective inhibitors of BH3-mimetics. Especially in cases of venetoclax used as monotherapy or in combination with other agents^24^. Xie et al.^25^ studied cardiac I/R injury and observed the Bcl-2 and Bax ration in cardiomyocytes. The authors found that Bcl-2 to Bax ration was decreased in I/R group, suggesting that Bcl-2 and Bax were increased in I/R group, but Bcl-2 decreased after reperfusion. Finally, they suggested that the marked increase in Bcl-2 outside the reperfused area may be a mechanism to rescue surviving cardiomyocytes.

In addition, Han et al.^26^ studied I/R in heart and analyzed the Bcl-2 and Bax expression. In I/R group, Bax expression was increased, and Bcl-2 expression was decreased compared to control group. They stated that, compared to the myocardial I/R model group, a significant decrease in cardiomyocyte apoptosis was observed in the gastrodin group (P < 0.05). They also reported that protein and mRNA expression levels were decreased for Bax and activated caspase-3 in the gastrodin group, while increased for Bcl-2 (P < 0.05). It has also been stated that gastrodin can reduce inflammatory cytokines (P < 0.05) and regulate anti-inflammatory cytokines such as interleukin-10 (IL-10) (P < 0.05) in the serum of Sprague Dawley rats. They concluded that gastrodin plays a protective role in myocardial I/R injury by regulating the expression levels of apoptosis-related signaling proteins and inflammatory cytokines.

In a study of cardiac ischemia reperfusion, Bcl-2 and Bax gene expression were analyzed. The authors found that Bcl-2 and Bax expression were increased in I/R group compared to control group, and this increase was significantly different. Compared to the control group, MDA content, myocardial apoptotic index, and Bcl-2, Bax protein expression were significantly increased in the I/R group, but MDA, myocardial apoptotic index content and Bax protein expression were significantly decreased. Based on the expression of the Bcl-2 protein, it was expressed up-regulated in the Ethyl pyruvate group (P < 0.05). These results concluded that Ethyl pyruvate was able to inhibit apoptosis of cardiac myocytes, possibly by attenuating oxidative stress, upregulating Bcl-2 and downregulating Bax proteins^27^.

In an experimental study comparing daidzein with genistein, it was stated that it did not support the events associated with endometrial cell proliferation and was seen as a compound with a potential safety profile. With this aspect, it was stated that more experimental studies are needed^28^. In a different experimental study, it has also been suggested that daidzein may improve ovarian oxidative stress and inflammation in pigs. This process was thought to be achieved by suppressing the TLR4/NF-κB signaling pathway and activating the Nrf2/HO-1 signaling pathway^29^. Daidzein has been used in experimental studies in recent years as a classical isoflavonic phytoestrogen with specific estrogenic activity.

Sirotkin et al.^30^ reported for the first time that daidzein affects the basic ovarian cell functions (proliferation, apoptosis, and hormone secretion) of this soy isoflavone and alters the effects of follicle stimulating hormone (FSH). They observed that daidzein promoted the effect of FSH on ovarian cell proliferation and apoptosis, and suppressed or even reversed the effect of FSH on hormone secretion. In conclusion, due to the direct effect of daidzein on basic ovarian cell functions and the ability of these cells to respond to FSH, soy-containing diets have a potential effect on female reproductive processes through a direct effect on the ovary.

Supporting this conclusion, another experimental study reported that dietary daidzein supplementation improved reproductive performance and fetal development in mice, which was associated with changes in serum hormones, tissue antioxidant capacity, and expression levels of reproductive genes in both mother mice and offspring^31^. Treatment with soy isoflavones has been noted to exert beneficial effects in polycystic ovary syndrome rats (with reduced aromatase activity), which may be due to their ability to reduce testosterone concentration in peripheral blood. Analysis of physical, biochemical, and histological evidence concluded that soy isoflavones may be beneficial for polycystic ovary syndrome^32^.

Another study supports this view. It has been observed that the combination of genistein and daidzein with Bacillus coagulans shows hormone restoration, reduces oxidative stress, improves menstrual cycle, and improves ovarian physiology. In addition, genistein and daidzein together with Bacillus coagulans have been reported to have a beneficial role in increasing the bioactivity of soy isoflavones. As a result, it has been suggested that higher doses of Bacillus coagulans (6.50 mg/kg) are more effective in improving the symptoms of polycystic ovary syndrome^33^.

## Conclusion

After the I/R procedure, pycnosis and apoptosis were observed in the cells due to increased degeneration and inflammation in the ovary, and Bax and Bcl-2 signal increased. After daidzein treatment, a decrease in Bax and Bcl-2 signal was observed due to the decrease in degeneration and inflammation in the germinal epithelium and pre-antral and antral follicles. It was observed that daidzein stopped the apoptotic process.

## Data Availability

All data sets were generated or analyzed in the current study.
